# Effect of Artificial Aging on Mechanical and Tribological Properties of CAD/CAM Composite Materials Used in Dentistry

**DOI:** 10.3390/ma14164678

**Published:** 2021-08-20

**Authors:** Marcel Firlej, Daniel Pieniak, Agata M. Niewczas, Agata Walczak, Ivo Domagała, Anna Borucka, Krzysztof Przystupa, Joanna Igielska-Kalwat, Wojciech Jarosz, Barbara Biedziak

**Affiliations:** 1 Department of Craniofacial Anomalies, Poznan University of Medical Sciences, Bukowska 70, 60-812 Poznan, Poland; marcel-firlej@wp.pl (M.F.); ivo.m.domagala@gmail.com (I.D.); joanna.igielska@wp.pl (J.I.-K.); biedziak@ump.edu.pl (B.B.); 2 Department of Mechanics and Machine Building, University of Economics and Innovations in Lublin, Projektowa 4, 20-209 Lublin, Poland; daniel.pieniak@wsei.lublin.pl; 3 Departament of Conservative Dentistry with Endodontics, Medical University of Lublin, W. Chodźki 6, 20-093 Lublin, Poland; agata.niewczas@umlub.pl; 4 Departament of Fire Technology the Main School of Fire Service, Faculty of Safety Engineering and Civil Protection, Slowackiego 52/54, 01-629 Warsaw, Poland; awalczak@sgsp.edu.pl (A.W.); wjarosz@sgsp.edu.pl (W.J.); 5 Faculty of Security, Logistics and Management, Military University of Technology, gen. S. Kaliskiego 2, 00-908 Warsaw, Poland; anna.borucka@wat.edu.pl; 6 Department of Automation, Lublin University of Technology, Nadbystrzycka 36, 20-618 Lublin, Poland

**Keywords:** dental materials, composites, CAD/CAM, 3D printing, artificial saliva, sliding wear, indentation hardness, coefficient of friction

## Abstract

With easy-to-process 3D printing materials and fast production, the quality of dental services can be improved. In the conventional procedure, the dentist makes temporary crowns directly in the patient’s mouth, e.g., from the most commonly used bis-acrylic composites. Temporary crowns made directly in the office without the use of CAD/CAM are often of inferior quality, which directly results in impaired hygiene, poorer masticatory mechanics, greater deposition of plaque, calculus and sediment, and may adversely affect periodontal and gum health. The mechanical strength, resistance to aging and abrasion of 3D printing materials are higher than those of the soft materials used in conventional methods. This translates into durability. The patient leaves the surgery with a restoration of higher utility quality compared to the conventional method. The objective of the paper was to determine the influence of aging in artificial saliva of AM (additive manufacturing) orthodontic composites on their functional properties. For the purpose of the study, fillings well-known worldwide were selected. These were traditional UV-curable resins (M I, M II, M III, M V) and a hybrid material based on a UV-curable resin (M VI). Samples were stored in artificial saliva at 37 ± 1 °C in a thermal chamber for 6 months. Indentation hardness, frictional tests and sliding wear measurements were conducted. A comparison between various materials was made. Descriptive statistics, degradation coefficients, H^2^E, Archard wear and specific wear rate were calculated. The Weibull statistical test for indentation hardness was performed and Hertzian contact stresses for the frictional association were calculated for unaged (M I, M II, M III, M V, M VI) and aged (M I AS, M II AS, M III AS, M V AS, M VI AS) samples. M I exhibited the lowest average hardness among the unaged materials, while M III AS had the lowest average hardness among the aged materials. Comparably low hardness was demonstrated by the M I AS material. The coefficient of friction values for the aged samples were found to be higher. The lowest wear value was demonstrated by the M I material. The wear resistance of most of the tested materials deteriorated after aging. The M VI AS material had the highest increase in wear. According to the results provided, not only the chemical composition and structure, but also aging have a great impact on the indentation hardness and wear resistance of the tested orthodontic materials.

## 1. Introduction

**Figure 1:**
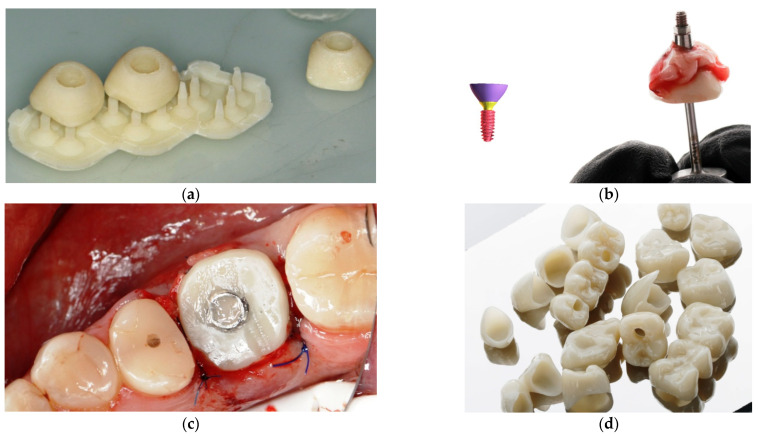

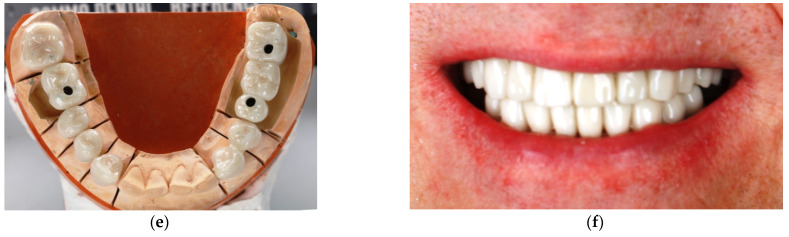
The process of manufacturing and applying an individual patient’s emergence profile: (**a**) 3D printout of an individual gingival emergence profile for an implant with supports, (**b**) cemented gingival emergence profile for an implant abutment, (**c**) individual healing screw cemented on a prosthetic abutment, screwed to the implant in the bone (**d**) 3D prints of temporary crowns after removal of the structural supports that have been polished and varnished (**e**) 3D printed full arch temporary crowns placed on the plaster model of the patient’s jaw, (**f**) the effects after cementing the temporary crowns in the patient.

Dental treatment is often multi-step in nature and requires long-term temporary restorations intended for use over a period of several weeks to several months. For example, temporary restorations are indicated for partially edentulous patients undergoing implant treatment when the teeth adjacent to the edentulous area are restored with crowns [1]. In such cases, temporary restorations prevent loading of newly placed implants or tissue grafts during the healing phase, which is the case with removable partial dentures. Temporary restorations are indicated to protect the dental pulp and periodontal tissues, prevent lateral tooth movement, help maintain a stable tooth position, and ensure adequate chewing efficiency and smile aesthetics [2]. Sample photos covering the treatment process using this method are presented in Figure 1. Treatment planning in the CAD/CAM environment enables the removal of the teeth, insertion of the implant in place of the tooth and the closure of the extraction socket with an individual healing screw, ensuring the desired emergence profile and the shape of the gingiva suitable for a given tooth (Figure 1a–c). Temporary crowns made by 3D printing are also used in minor prosthetic rehabilitation of the patient’s masticatory organ. The patient can adapt to the planned shape and cavity of the teeth with easy-to-manufacture 3D-printed crowns, which is important in the case of rehabilitation of the entire masticatory organ and occlusal reconstruction (Figure 1d–f). In addition, this solution allows the patient to function and bite normally immediately after the procedure while the dental implants heal, which usually takes 4–9 months. The patient does not have to wear uncomfortable removable dentures of vastly inferior function and comfort compared to permanent restorations attached to the implants (Figure 1f). Temporary restorations have also become an effective diagnostic tool for evaluating the effectiveness of shape matching, as well as for checking and testing occlusion and periodontal response, and for checking the progress of healing after implantation. Specific applications of temporary restorations in dentistry include:

1. Cases of major prosthetic and orthodontic-prosthetic rehabilitation. Here, a thorough diagnosis preceded by taking diagnostic impressions and determining the therapeutic position is necessary. The therapeutic position determines the mutual positioning of the teeth to stabilize the mandibular alignment in all planes. This requires long-term use of temporary crowns or onlays (with or without tooth preparation) to reprogram the masticatory muscles and adapt the temporomandibular joint to its new normal position. These can be cemented permanently or used as removable restorations such as bite splints worn around the clock or for a required number of hours. This type of restoration is also used in the treatment of MLD (mandibular lateral displacement/deviation), in the repositioning of the TMJ disc, in the treatment of asymmetrical or symmetrical compressions and distractions of the temporomandibular joint.
2. Cases of immediate loading of implants. By making temporary crowns for the entire dental arch before the removal of old teeth, it is possible to prepare temporary crowns using the CAD/CAM method even before implantation on the basis of photographs and the patient’s smile design. Temporary crowns can be immediately screwed on and are used in this form for at least 5–9 months. After a period of healing, they are replaced with the final restoration using the immediate implant bridge.
3. Cases of patients who undergo gingivectomy (gum resection). In these cases, temporary crowns or veneers should be used for a period of 2–4 months until the shape of the gums stabilizes, before the final dental restoration is performed.
4. Cases of patients who receive dental implants followed by individualized gum emergence and shaping profile using individual shapes generated in software and screwed to the implant for 1–9 months.

Temporary restorations are often used in the oral cavity for a period of several months, which requires high durability of the restoration [3]. As demands from patients and dentists increase, new surgical materials and techniques are developed.

In recent years, CAD-CAM additive technology including 3D printing has been increasingly used for the fabrication of temporary crowns. Digital light processing (DLP) composite materials have been widely used in this area [4,5,6]. The DLP 3D printing technique involves curing polymer composites with the light of a projector or an LCD screen. Due to the good quality of the reproduction of the nominal dimensions of the CAD model by the printout, and the low roughness of the printed model, the DLP technology is now increasingly used in dentistry to make extremely accurate and efficient reconstructive elements. Such models and devices are particularly applicable in orthodontics and prosthetics [7,8,9,10,11]. Special polymer composites are used as structural materials for temporary crowns and orthodontic splints in particular [12,13].

Temporary restorations in the oral setting are exposed to aging factors. One of the most important factors is the humid environment, in particular due to the influence of saliva and food, as well as a relatively constant temperature. Some believe that in the structure of polymer matrix composites, water acts as a plasticizer and leads to stress relaxation and reduction of material stiffness [14]. However, the mechanism of hydrolytic degradation has not been sufficiently investigated. It is likely that chemical compounds in aqueous solution cause decomposition of the polymer network by breaking down ester bonds [15]. Degradation of the polymer network depends on the presence of specific chemical groups in the molecular structure, such as ester, amide, and urea groups [16]. The intensity of material degradation also depends on the surface condition of the restorations as well as the type and proportion of additional substances (filler particles, type of initiators) [16,17].

There are documented cases of clinical failure due to the effect of humid environment on complex CAD/CAM polymeric materials [18]. Due to the complexity of the problem of hydrolytic aging, and even more so the impact of artificial saliva of complex chemical composition, it is not possible to analytically predict the degradation of functional properties of complex CAD/CAM materials. The clinical success of a material depends largely on its behavior under mechanical load and tribological processes when exposed to oral cavity conditions. Clinical studies are not yet available and in vitro studies may provide new information for clinical applications [19]. However, the results of clinical trials show no differences in the degradation of composite materials of different structures [20]. Comparing test results obtained in different units is also difficult. Moreover, superficial observations often lead to premature interpretations, mainly due to the dynamic behavior of durability curves [21]. In contrast, laboratory studies can focus on individual degradation mechanisms. Therefore, mechanical and tribological properties can be determined experimentally according to standardized laboratory methods for material compositions and fabrication methods independently. It was assumed that degradation at the biotribological junction does not depend only on contact stresses, but also on hydrolytic aging and chemical corrosion. For example, salivary enzymes or acids may increase wear of restorative materials through corrosive processes [22]. Tribological wear may be associated with non-tribological wear [23,24] and other surface layer effects, whose influence on the properties of the surface layer of dental polymer composites is not obvious [24], e.g., in the paper by [25], an increase in nanoindentation hardness of composites was observed after exposure in a humid environment with physiological temperature. Therefore, unlike many other papers, tribological tests before and after artificial aging were assumed but in conditions taking into account the lubrication mechanism of saliva [26] and physiological temperature. Practical confrontation of the obtained results with those obtained by other researchers is difficult, therefore wear rates of practical importance, hardness to modulus of elasticity [27] and Archard wear [28] and specific wear rate [29] can be used. In the light of the above analysis of the problem, the aim of the paper was formulated. It was assumed that the purpose of this study is to evaluate the long-term effect of artificial saliva and temperature simulating physiological oral cavity conditions on the surface of DLP 3D printing materials used in the fabrication of temporary dental restorations. The null hypothesis is that they have no effect on the microindentation properties and the sliding friction and wear of resin-based DLP 3D printed CAD/CAM materials aged in artificial saliva for up to 6 months.

## 2. Materials and Test Method

### 2.1. Materials

Five materials dedicated for additive 3D printing applications in the dental industry were used in this study. These were traditional UV-curable resins (M I, M II, M III, M V) and a hybrid material based on a UV-curable resin (M VI). The chemical composition characteristics of the polymers used according to the manufacturers data are shown in Table 1.

### 2.2. Sample Preparation and Aging

The samples were made in the shape of cylindrical discs 30 mm in diameter and 6 mm thick and cubes 6 mm thick, 25 mm wide and 25 mm long. Samples of similar sizes were used in [36]. Table 2 and Table 3 show the printing parameters of the samples for each material. All samples were sprayed with Isopropyl alcohol (IPA) for 1 min. to remove excess resin before final rinsing in Anycubic Wash & Cure. All printed samples were rinsed in 99.9% isopropyl alcohol to remove any residual uncured resin, followed by exposure in an Anycubic All-in-one Wash & Cure machine (Shenzhen, China) (Table 4 and Table 5) [37]. The samples were then ground (P600, P1200 and P2400 grit) and polished with honing disks and diamond slurry (1 μm) on a Saphir 550 grinding and polishing machine (ATM Gmbh, Mammelzen, Germany). In the next step, the samples were rinsed (washed) in water. The control group consisted of samples not conditioned in artificial saliva. A total of 10 disc-shaped and 10 cubical samples were made from each material. Once made, the samples, from each material separately, were stored in a tank with saline at room temperature for 7–10 days. Then half of the samples—five discs and five cuboids—were subjected to indentation and sliding wear tests. The other half of the samples were conditioned. This part of samples were immersed in artificial saliva (pH = 5.3) and aged (stored) for 6 months in conditions simulating those of the oral environment in a Q-Cell laboratory thermal chamber (Pol-Lab, Wilkowice, Poland) at 37 °C. The composition of the artificial saliva was prepared based on the PN-EN ISO 10271:2012 standard [38]: NaCl—0.4 g, KCl—0.4 g, NaHO_4_ x H_2_O—1.35 g, NaH_2_P—0.78 g and Na_2_S x 9H_2_O—0.005 g in 1 dm^3^ of solution. The conditioned samples were then subjected to indentation and sliding wear tests.

### 2.3. Indentation Hardness Measurements

A Micro Combi Tester universal microhardness tester (MCT, Anton Paar GmbH, Ostfildern, Germany) was used to measure the mechanical and elastic surface parameters of the tested samples. The number of samples in each test group was 5 (n = 5). Indentation hardness *H_IT_* was determined as the ratio of the highest normal force loading the indenter *P_max_* to the contact area of the indenter under maximum load *A*, according to formula (1) [39]:

(1) $$H_{IT}=\frac{P_{max}}{A}$$

Stiffness *S* was determined from relationship (2) [39]:

(2) $$S=\frac{dP}{dh}=\beta \cdot \left(\frac{2}{\sqrt{\pi }}\right)\cdot E^{*}\sqrt{A}$$

**Figure 2:**
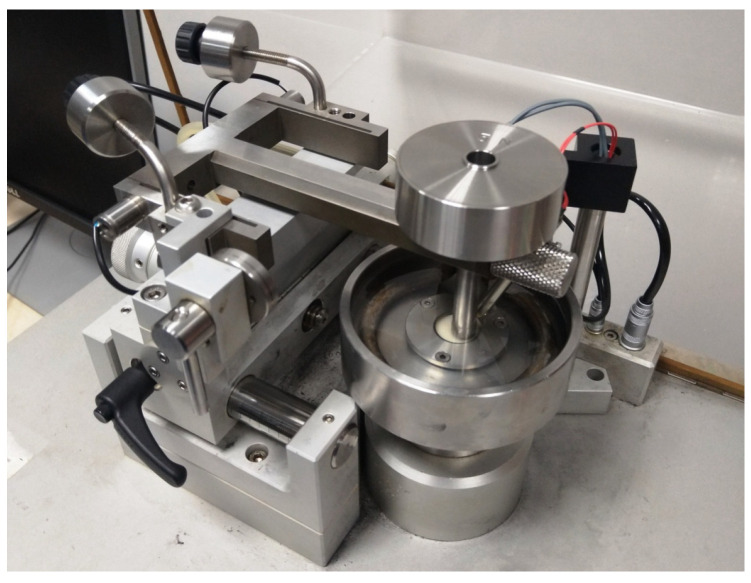
Ball-on-disc setup for wear testing.

In Equation (2) the relationship $\frac{dP}{dh}$ was determined from the indenter force-displacement diagram (Figure 2). Parameter *β* is equal to 1 for a symmetric indenter and for a Vickers indenter the corrected value of the *β* parameter is *β* = 1.0055 [39].

The value *A* is a function of the depth *h_c_* and is determined from relationship (3), according to [40]:

(3) $$A=F\left(h_{c}\right)\;=24.54h_{c}^{2}+C_{1}h_{c}^{1}+C_{2}h_{c}^{1/2}+C_{3}h_{c}^{1/4}+C_{4}h_{c}^{1/8}+\ldots +C_{n}h_{c}^{1/2n}$$

In Equation (3), the calculations are based on the constant *C_n_*, which expresses the indenter geometry. The method of determining the constant *C_n_* is described in [41]. In stiffness calculations, there is a surface layer modulus denoted by the letter *E*. This quantity is defined by Equation (4):

(4) $$\frac{1}{E^{*}}=\frac{1-\nu^{2}}{E}+\frac{1-\nu_{i}^{2}}{E_{i}}$$

In Equation (4), E* stands for the reduced modulus of elasticity and *ν* stands for the Poisson number. The value of E* takes into account the elastic deformations in both the tested sample and the indenter. This issue is further described in [39]. In contrast, *E_i_* and *ν_i_*, respectively, apply only to the indenter. The relationship (4) is a general relationship that applies to any symmetric indenter, not limited to a particular geometry such as a cone or pyramid. The Equation (4) was originally introduced for the case of elastic contact; however, it has also proven to be appropriate for the case of elastic-plastic contact [39].

Based on the stiffness *S* calculated from the Equation (2) and the penetration path of the indenter corresponding to the elastic deformations of the test surface—*h_c_*, the indentation modulus of elasticity *E_IT_* was calculated according to Equation (5):

(5) $$E_{IT}=\frac{\sqrt{\pi }S}{2\beta \sqrt{A\cdot hc}}$$

### 2.4. Sliding Wear

Sliding friction tests were performed using a universal microtribometer (CSM Instruments SA., Peseux, Switzerland), in a ball-on-disc setup (Figure 2). The samples were tested in artificial saliva at a constant temperature of 37 °C. Cylindrical discs made of 3D printed materials were used as samples, while the counter-samples were beads with a diameter of Ø 6 mm. The beads were made of aluminum oxide (Al_2_O_3_). The material and diameter of the counter-sample were selected based on our own experience, according to the principles reported in [30]. During the test, the bead was stationary while the disc rotated at a frequency of 1.5 Hz. A constant load of 5 N was applied. The number of samples in each test group was 5 (n = 5). The course of the coefficient of friction μ as a function of the number of load cycles was recorded. After the friction tests, cross-sectional profilographs of the wear tracks were analyzed. Profile measurements were carried out with the use of Dektak 150 surface profilometer (Veeco, Plainview, NY, USA). All tested samples were measured on the plane transverse to the sliding direction. Distance between profiles was about 35 degrees, 10 measurements were made around the perimeter of the wear scar.

### 2.5. Statistical Analysis

The statistical analysis aimed to evaluate the individual measurement results in the analyzed groups of materials and the relationships between them. Basic descriptive statistics were determined for general evaluation first. The results were grouped according to the material tested and the “before and after aging” category of the sample. For the results of the indentation test the mean value (Mean), standard deviation (Std. Dev.), minimum (Min.) and maximum (Max.) values, sample size (N), and median (Median) are calculated and reported. One of the main factors affecting surface strength is the reproducibility of material hardness [42]. The statistical scatter of the hardness *H_IT_* measurement results was evaluated using Weibull analysis. The purpose of the analysis was to determine the scale and shape parameters of the Weibull distribution. The scale parameter defines a characteristic hardness value, which corresponds to 63.2% of the cases in the considered population. The shape parameter (Weibull modulus) can be considered as an indicator of the uniqueness of surface hardness. The Weibull analysis procedure has been described in, inter alia, [43,44]. For the results of the sliding wear test, the box plot shows the mean value, confidence interval and standard deviator. In order to identify significantly different groups for non-normal distributions, the Wilcoxon Rank-Sum Test was used, which is a non-parametric alternative to the multiple Student’s *t*-test. The Wilcoxon Rank-Sum Test is used when a variable is measured several times. The test examines the difference between pairs of measurements of the tested characteristic for each of the tested objects. This difference is used to verify the null hypothesis of equality of medians. Statistica 13 software (StatSoft, Tulsa, OK, USA) was used for statistical calculations.

## 3. Results

### 3.1. Indentation Hardness, Stiffness and Elasticity

Descriptive statistics of the results of the indentation hardness measurements are shown in Table 6a,b.

**Figure 3:**
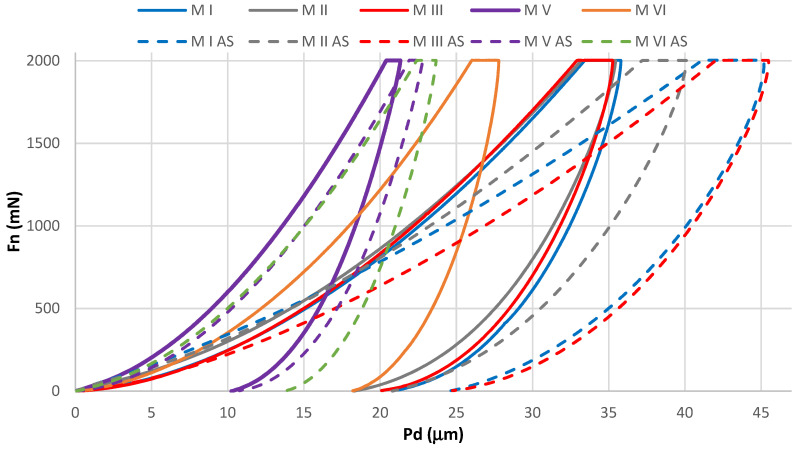
Mean of characteristics, normal load (*Fn*)—penetration depth (*Pd*); unaged samples M I, M II, M III, M V, M VI, aged samples M I AS, M II AS, M III AS, M V AS, M VI AS.

For unaged (M I, M II, M III, M V, M VI) and aged (M I AS, M II AS, M III AS, M V AS, M VI AS) samples, the average waveforms of the “normal force-indenter penetration depth” characteristics are presented in Figure 3. The Wilcoxon statistical test showed significant differences in the comparisons of *H_IT_* results of unaged and aged samples. Values of the p = 0.000012 statistics were shown in comparisons in four of the five material groups. In the comparison between M V and M V AS groups, values of the p = 0.000016 statistics were shown. A summary of the Wilconox test results is presented in the Appendix A (Table A1, Table A2 and Table A3). In the hardness analysis of the unaged samples, the M V material demonstrated the highest average hardness *(H_IT_*). The same material exhibited the highest hardness after aging. The lowest average hardness among the unaged materials was demonstrated by M I, while among the aged materials, M III AS exhibited the lowest average hardness. Comparably low hardness was demonstrated by the M I AS material.

In the *S* stiffness analysis of the unaged samples, the highest value was observed for the M VI material. The aging process caused a significant decrease in its stiffness. The M VI AS material retained the highest stiffness in the group of aged materials.

In the *E_IT_* indentation modulus of elasticity analysis, the material properties were found to vary. A significant decrease in *E_IT_* modulus after aging was observed for all materials tested. The lowest average modulus of elasticity among the unaged samples was demonstrated by the M II material, and after aging—by the M I AS material. The mean values of the modulus of elasticity of materials M I, M II and M III were similar.

The reduced modulus of elasticity E* varied due to aging as did the indentation modulus. Differences in the values of this parameter were also confirmed by the Wilcoxon statistical test. Value of the p = 0.000012 statistics was shown in comparisons of all groups determined by the material. The highest average E* value for unaged samples was observed for M VI material and in case of aged samples for M VI AS.

### 3.2. Elastic Deformation to Failure H^2^/E

**Figure 4:**
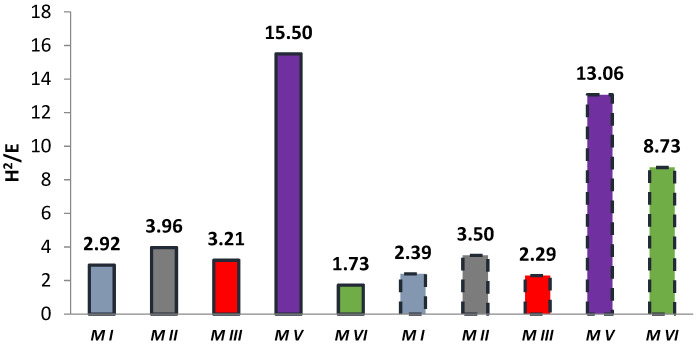
Summary of average coefficient *H*^2^*/E* values (solid bar edges—unaged samples, dashed bar edges—samples after aging).

In some papers, researchers use the *H*^2^/*E* coefficient defined by the ratio of the square of the indentation hardness *H*^2^*_IT_* to the modulus of elasticity E* [39,40,41,45]. This coefficient combines the ability to maximize elastic deformation (low modulus of elasticity) and the ability to minimize permanent deformation (high hardness). The *H*^2^*/E* coefficient expresses “elastic deformation to failure”. The literature reveals that the higher the *H*^2^*/E* value, the higher the wear resistance of the material [46,47]. The results of the *H*^2^*/E* coefficient calculation are shown in Figure 4. Among the tested materials, the highest value of the *H*^2^*/E* coefficient was demonstrated by the M V material, both before and after aging. Most of the aged samples had a lower *H*^2^*/E* coefficient value than the corresponding unaged samples. Only for the M VI material did the coefficient increase after aging. It should be noted that the M VI material had the lowest “to failure” ratio in the unaged group of samples. However, the modulus of elasticity E* did not change significantly, while the hardness of *H_IT_* increased by about 50%.

### 3.3. Indentation Hardness Repeatability—Weibull Analysis

The results of the shape and scale parameter calculations are presented in Table 6a,b.

The highest values of the shape parameter, indicating the lowest statistical scatter of hardness were obtained for M I, M II, M III materials without aging, and the lowest values were obtained for samples for M V and M VI materials without aging. In contrast, for the aged samples, this relationship was reversed. The M I AS, M II AS and M III AS materials had the lowest shape parameters, while the M V AS material had the highest shape parameter value. The largest value of the scale parameter was obtained for the M V material without aging. The scale parameters of M I, M II and M III materials without aging were comparable, but significantly lower than the M V material. After aging, the scale parameter values decreased significantly, except for material M VI, for which an increase was recorded.

### 3.4. Analysis of Elasticity Changes

In general, the degree of degradation in terms of material stiffness can be determined using the formula presented in [48] having the following form:

(6) $$\frac{dE\left(n\right)}{dn}=-Acn^{c-1}$$

where:

*A, c*—constants depending on material,

*E(n)*—residual stiffness,

*n*—number of load cycles (time).

Another way of calculating the degree of degradation based on the change in tangent modulus of elasticity, according to [49], may also be of practical significance

(7) $$D=1-\frac{E_{after\;aging}^{*}}{E^{*}}$$

where:

*D*—degree of degradation,

$E_{after\;aging}^{*}$—mean value of the tangent modulus of elasticity after aging,

E*—initial mean value of the tangent modulus of elasticity.

**Figure 5:**
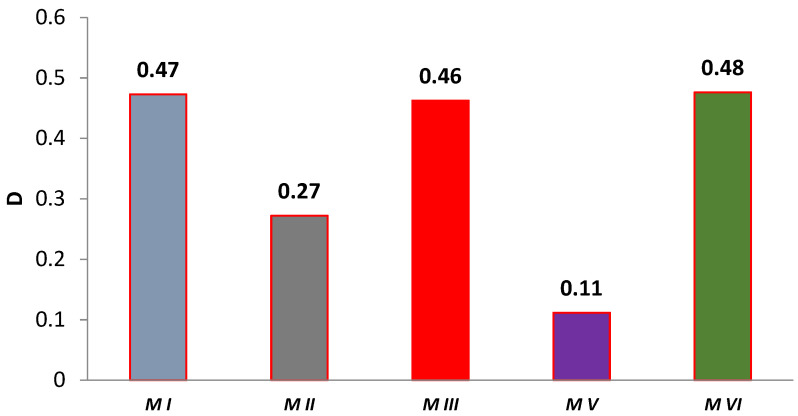
Graph of the degree of change of aging-dependent modulus of elasticity in artificial saliva.

Based on Equation (7), the material degradation rate was calculated based on the change in elasticity. The results of the calculations are shown in Figure 5. The degradation of the modulus of elasticity was found to be selectively differentiated and material dependent. The group of M I, M III, M VI materials stands out here and is internally comparable. The M V composite has the lowest degree of degradation. It is also the hardest of all the materials tested and is characterized by the values of the *H*^2^*/E* coefficient.

### 3.5. Results of Sliding Wear Tests

**Figure 6:**
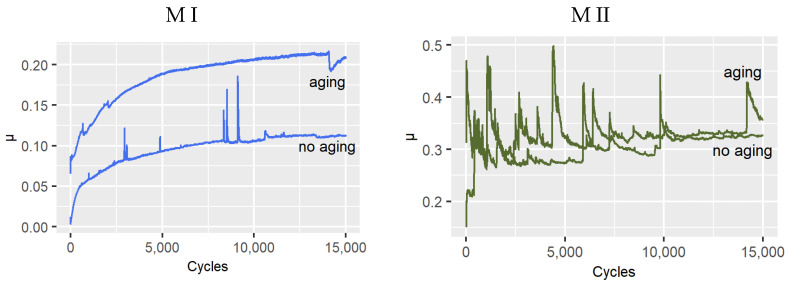

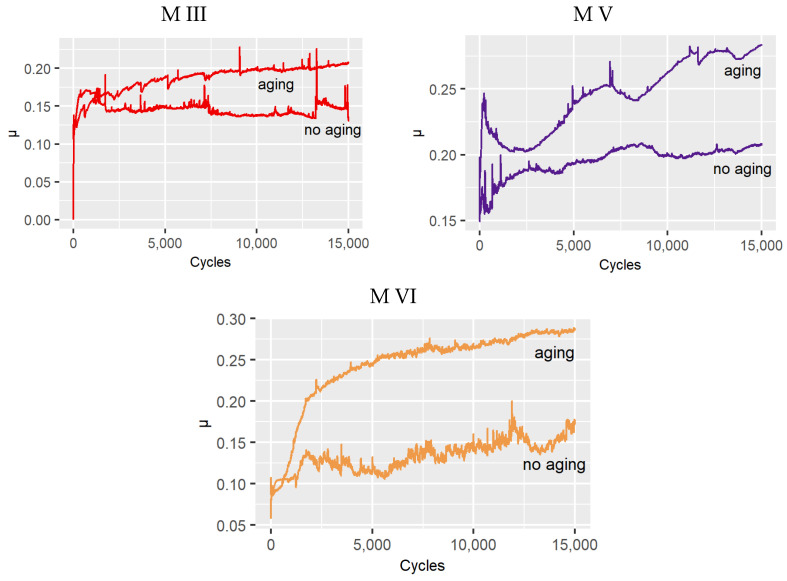
Dependence of the coefficient of friction on the number of friction cycles recorded in the ball-on-disc test.

In friction tests in artificial saliva, the variation patterns of the sliding coefficient of friction were recorded. Figure 6 summarizes friction representative plots depending on material and effect of aging in artificial saliva. The coefficient of friction values for the samples after aging were found to be higher. Only for the M II material were the differences in coefficient of friction values found to be relatively small.

**Figure 7:**
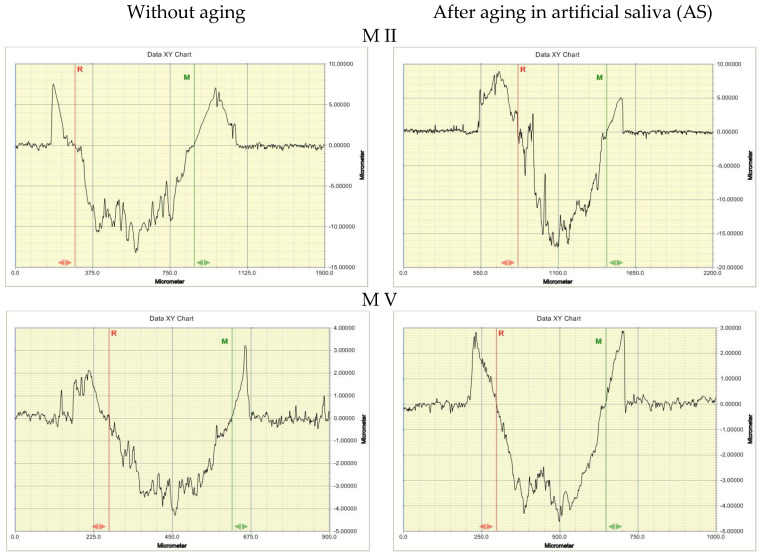

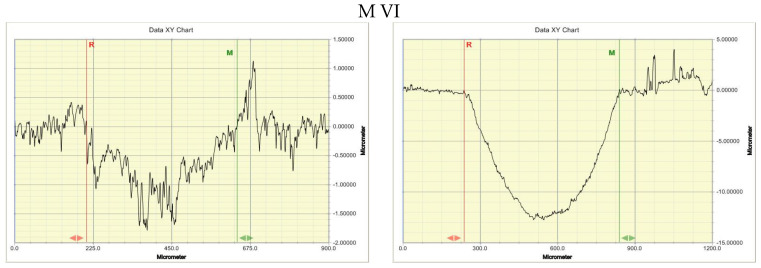
Profilographs of wear track cross-sections of the tested materials in abrasion test.

**Figure 8:**
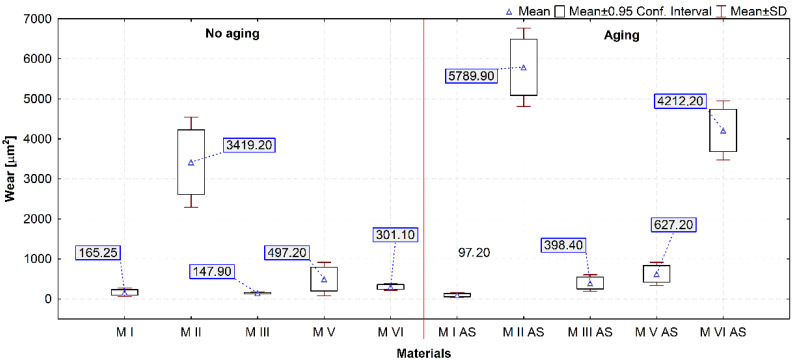
Box plot of the cross-sectional area of the wear track (AS—samples aged in artificial saliva).

After friction testing, wear tracks were checked using a contact profilometer. The cross-sectional area of the wear track was used as a measure of wear. Figure 7 shows selected profilographs for materials exhibiting diverse wear behavior. Figure 8 presents a box plot of the results of the statistical calculations of wear of the tested materials.

The highest average wear value expressed by the cross section of the wear track (Figure 8) was demonstrated by the M II material, for both aged and unaged samples. The lowest wear value was demonstrated by the M I material. The wear resistance of most of the tested materials deteriorated after aging. The M VI material exhibited the highest increase in wear after aging. This is also confirmed by the Wilcoxon test results for this material (*p* = 0.005062). For the M I material wear remained unchanged (*p* = 0.059337). Also in the case of the M V material, no differences were found in the statistical Wilcoxon test. It should be noted that a high level of wear of the M II material compared to the M I and M III materials was found, despite the similar properties determined in sample indentation. It can be assumed that this is due to the different chemical structure of the M I and M III materials compared to the M II material.

### 3.6. Analysis of the Effect of Material Hardness on Tribological Properties

In dentistry, many kinematic junctions require polymer composites with low Archard’s wear coefficient *K*. The Archard coefficient is the product of the wear rate and the material hardness [50]. According to Borrero-Lopez et al. [51] the range of Archard wear coefficient can serve as a basis for quantitative prediction of the life of wear-limiting dental materials. The results of calculating the values of these coefficients and the Hertzian contact stress are presented in Table 7.

The Archard wear coefficient was calculated from Equation (8) [52]:

(8) $$K=\frac{H\cdot V}{F_{N}\cdot L}$$

where:

*H* (*H_IT_* in Table 6)—Vickers hardness of the composite [MPa],

*V*—volume loss of the composite due to wear [mm^3^],

*F_N_*—normal force [N],

*L*—friction path [mm].

In Table 7, the results of specific wear rate *k* calculations are additionally presented. Rate *k* was calculated based on Agarwal’s equation [53].

(9) $$k=\frac{V}{F_{N}\cdot L}$$

where:

*V* —volume loss of the composite [mm^3^],

*F_N_*—normal force [N],

*L*—friction path [m].

Hertzian contact stress was calculated according to the formulas presented in [54].

Archard’s wear coefficient *K* and specific wear rate *k* are varied. Higher values of both coefficients prove to be unfavorable. The highest coefficient *K* value for samples before aging was found for the M VI material and, in case of aged samples, for the M I material. The highest value of coefficient *k* among unaged samples was calculated for the M V material. In case of aged samples, the *k* value was calculated for the M VI material. This material had relatively high values of coefficient k both before and after aging. In general, the most favorable values of both coefficients were found for the M I material.

## 4. Discussion

### 4.1. Indentation Properties

Cyclic sorption and desorption of water induces internal mechanical stresses in polymer composites, strong enough to initiate formation of microcracks. The effect of water is more destructive in case of self-stress in the material structure. Moisture absorption in materials of this type depends on: matrix type, exposure time, temperature, shape of the element, relative humidity and lighting conditions [55]. In general, the process of diffusion of water molecules into multicomponent materials can be described by Fick’s law, according to which the diffusion rate is dependent on time and temperature [56]. The mechanism of hydrolytic degradation is not yet fully understood. It is likely that chemical compounds in an aqueous solution cause decomposition of the polymer network by breaking down ester bonds [15]. It is also believed that moisture absorption depends on the amount and type of filler particles and the type of monomers used to form the matrix [57]. The intensity of material degradation also depends on surface condition, structural defects as well as the type and proportion of additional substances (e.g., filler particles, initiators) [16]. The thesis [58] demonstrated the effect of moisture absorption on the decrease in flexural strength of materials used to make bite splints. In theses [59,60], the influence of moisture on changes of hardness of conventional dental restoration composites was shown. In the thesis by Rayyan et al. a higher water absorption was observed in traditional composites than in CAD/CAM composites [61].

In the present thesis, the problem of the effects of moisture on the composite structure in additive CAD/CAM technology was evaluated similarly to the theses [62,63]. The hardness of the materials tested in this study was shown to be relatively low compared to standard reconstruction composites. The mechanical properties of the tested materials were found to change differently during aging. Three materials—M I, M II and M III had similar hardness before aging. On the other hand, after aging, the hardness of M II, a material with structure different from M I and M II, decreased to a lesser extent. The degradation rate of this material was also lower. It should be noted that M II is a material without inorganic fillers. Most remarkable is the increase in hardness of the M VI material after aging, which was accompanied by a significant decrease in modulus of elasticity. This is probably due to the way these parameters are determined according to Oliver&Pharr [39]. In this case, the elasticity test results translate into a relatively high value of degradation coefficient *D*. The increase in coefficient *H*^2^*/E* of the M VI material after aging, caused mainly by a significant increase in indentation hardness, may be related to its structure. It is the material with the most complex composition among the tested materials, classified as a micro-hybrid material with microfiller [35]. This phenomenon can be explained based on the artificial aging time adopted in the study. Drummond et al. [64] found that when water is absorbed into the resin matrix, the composite becomes more flexible. This can cause a sharp increase in some of its mechanical properties. However, over time, leaching of the components as well as swelling and degradation of the crosslinked matrix and hydrolysis in the interphase area eventually leads to a decrease in mechanical properties [65]. Such a case was analyzed in [59]. The aging rate of polymer composites depends mainly on their physical and chemical properties. Amorphous polymers are more susceptible to hydrolytic degradation than crystalline materials, linear polymers to a greater extent than branched polymers with higher molecular weight. Degradation rate depends on the presence of specific chemical groups in the molecule, such as ester, amide, and urea groups [16]. The intensity of degradation also depends on the surface condition of the material, defects found in it as well as the type and proportion of additional substances (e.g., filler particles, initiators) [16].

### 4.2. Friction and Abrasive Wear

One cause of abrasive wear of the dentition is attrition [24]. Attrition occurs as a result of contact and interaction between opposing teeth [66]. The level of attrition progression is mainly influenced by occlusive forces, as well as frictional contact geometry and oral cavity environment conditions [67]. Restrained or loose abrasive particles may be present in the frictional contact zone [68]. If both contact surfaces are hard and brittle at the same time, then deformation of surface micro-roughness occurs, followed by micro-cracking when the critical stress value is exceeded. When, in a frictional contact, the surface of one body has a significantly higher hardness than the antagonistic surface, rapid wear occurs by micro-attrition [69]. As reported in [70], attrition wear of the enamel surface can be up to 40 µm per year. This type of wear occurring in the process of mastication alone is quite low. On the other hand, it is often the result of a disease—bruxism [71]. The paper [72] describes a case in which bruxism caused the clenching of 22 maxillary teeth with a force of 50–200 N per night (7 h of sleep). Maximum occlusive forces in patients with bruxism range from 450 to 650 N [73]. The average forces can be 120 N [74] or 380 N [75]. The researchers found that deformation and wear of prosthetic crown surfaces are also observed at the occlusive contact points of the teeth. New materials used in 3D printing technology must exhibit similar wear resistance to conventionally used materials [76]. Lower wear resistance can lead to reduced stability of occlusive contacts due to premature formation of defects on abraded surfaces or even to shortened life of the entire temporary crown [77]. Tooth restorations should be abrasion resistant and prevent abrasion of opposing contact surfaces. According to [36], there is little research into abrasion resistance of occlusion devices.

In this study, the tribological parameters investigated were the coefficient of friction and wear resistance before and after aging the samples in artificial saliva. Wear coefficients were determined. Most of the materials tested (except M VI) exhibited deterioration of the tribological properties of the surfaces after aging in artificial saliva at oral cavity temperature. In the group of materials tested, the tribological properties of the surface layer changed to varying degrees during aging. Similar to theses [77,78], it was confirmed that the wear resistance of a material is related to its hardness. However, it was noted that the hardest and most rigid materials, M V and M VI, were not found to be the most resistant to wear when aged in artificial saliva.

The results of tests presented in this paper reveal the influence of artificial aging on the variation of the coefficient of friction. The highest variation in the coefficient of friction before and after aging was observed for the M VI material. The lowest variation was observed for the M II material (Figure 6). The interrelationships in biotribological nodes may be variable during friction [79], which is confirmed by the results obtained. In the study of dental composites, it was occasionally observed that the coefficient of friction increases along with wear due to the intensification of mechanical component of friction [80]. But such a relationship occurs in the absence of associated effects [81]. Also, a change in surface roughness can cause a change in the coefficient of friction, but the lubricating effect of artificial saliva is also of importance. The salivary pellicle has an effect on the coefficient of friction [82]. However, in case of the M VI material, a significantly higher wear after aging was demonstrated, which may indicate the importance of the described factors. Moreover, the M VI material as a microfilling material contains larger filler particles. It was reported in [83] that such a proportion of these particles in the composite structure causes an increase in the coefficient of friction. But it seems that the increase in the coefficient of friction after aging was related to the exposure of the filler particles.

It is possible that there was degradation of the polymer phase on the surface of the M VI material. In the literature, the mechanisms of degradation of polymer composites containing powder fillers by water particles: diffusion into the polymer network and hydrolysis reaction. In the first mechanism, water particles diffuse into the polymer network and fill the empty spaces between the polymer chains and micro-gaps. This phenomenon leads to plasticization and swelling of the polymer matrix. Cleavage of the polymer chains occurs, followed by leaching of the monomer. In the second mechanism, the hydrolysis reaction breaks down the bonds between the polymer phase and the filler, which can lead to filler separation [84]. On the other hand, an adequate range of the coefficient of friction is necessary to retain the patient’s physiological functions. As reported in [85], excessive values of the coefficient of friction may lead to premature wear, while overly low values may lead to reduced efficiency in crushing food.

The M I and M III materials demonstrated the highest resistance to wear before and after aging in artificial saliva for 6 months. But it should be noted that resin-based materials with additives can become brittle after an extended period of time [63], which does not occur in preclinical accelerated aging tests [36]. The paper [36] advocates the requirement of long-term clinical in vivo testing to confirm the results in terms of wear resistance [36]. However, according to the authors, the potential for clinical trials in this area is almost exclusively qualitative and not quantitative. The limitations of clinical trials relate to the study of wear mechanisms. Therefore, clinical trials should be supported by long-term in vitro studies under simulated laboratory conditions, taking into account loads and oral environmental factors.

The limitation of this study is its scope covering one of the many degradation mechanisms of DLP 3D printing dental materials, namely artificial aging. This compromises the methodical reproducibility of the study and has to be considered a limitation as the tests were conducted using artificial saliva made according to a technical standard. In addition, the effect of artificial aging was studied only with regard to surface properties. Aging times were limited. The researchers intend to continue the study after a longer exposure time in artificial saliva. In addition, it was noted that hydrolytic aging under non-stationary temperature conditions can be accelerated. The authors plan further research in this area.

## 5. Conclusions

The research described in this article investigates the effects of long-term storage in artificial saliva on the indentation hardness and abrasive wear of composite materials used for temporary CAD/CAM prosthetic and orthodontic restorations. Five commercial light-cured polymer composites used in dental practice for reconstructive work by 3D printing were tested.

Indentation hardness changes were determined. The degradation coefficient of the modulus of elasticity and the change in the coefficient of friction and abrasive wear resistance were determined. Storage in artificial saliva at oral cavity temperature was found to have a significantly negative effect on most indentation tested maintenance properties of the tested materials, except the composite with microfiller—M VI. In the case of sliding wear resistance, the M I material proved to be an exception. However, for this material the surface modulus of elasticity behaved differently, which resulted in the most unfavorable (highest) value of degradation coefficient. Unfortunately, the M VI material is characterized by the highest specific wear rate after aging. In case of resistance to sliding wear the M I material proved to be an exception. Archard’s coefficient and specific wear rate were found to be lower after aging, which should be considered as a very advantageous feature of the material. The effect was considered to have a nature of hydrolytic aging associated with changes in the structure of the materials.

Due to the results obtained, the null hypotheses can be largely rejected.

## Figures and Tables

**Table 1:** Characteristic and composition of additively manufactured polymers used for tests [30,31,32,33,34,35].

Material Brand	Producer	Name of Material in the Article	Printer	Chemical Composition	Concentration [% *w*/*w*]
Gr-17.1 temporary It	Pro3dure	M I	ASIGA UV MAX	7,7,9(or 7,9,9)-trimethyl-4,13-dioxo-3,14-dioxa-5,12-diazahexadecane-1,16-diyl bismethacrylate	20–50
				3,6,9-trioxaundecamethylene Dimethacrylate	10–25
				Phenyl bis(2,4,6-trimethylbenzoyl)-phosphine oxide	<50
				Silicon dioxide	<2
GR-10 guide	Pro3dure	M II	ASIGA UV MAX	Phenyl bis(2,4,6-trimethylbenzoyl)-phosphine oxide	≤2
				Esterification products: of 4,4′-isopropylidenediphenol, ethoxylated and 2-methylprop-2-enoic acid	≥95
GR-17 temporary	Pro3dure	M III	ASIGA UV MAX	7,7,9(or 7,9,9)-trimethyl-4,13-dioxo-3,14-dioxa-5,12-diazahexadecane-1,16-diyl bismethacrylate	20–50
				3,6,9-trioxaundecamethylene Dimethacrylate	10–25
				Phenyl bis(2,4,6-trimethylbenzoyl)-phosphine oxide	<50
				Silicon dioxide	<2
NextDent SG Orange	NextDent	M V	Phrozen Shuffle Lite 3D	Ethoxylated bisphenol A dimethacrylate	≥75
				7,7,9(or 7,9,9)-trimethyl-4,13-dioxo-3,14-dioxa-5,12-diazahexadecane-1,16-diyl bismethacrylate	30–50
				Phenyl bis (2,4,6-trimethylbenzoyl)-phosphine oxide	<10
NextDent C & B MFH	NextDent	M VI	Phrozen Shuffle Lite 3D	7,7,9(or 7,9,9)-trimethyl-4,13-dioxo-3,14-dioxa-5,12-diazahexadecane-1,16-diyl bismethacrylate	50–75
				2-hydroxyethyl methacrylate	<25
				Silicon dioxide	1–5
				diphenyl(2,4,6-trimethylbenzoyl)phosphine oxide	1–5
				Ethoxylated bisphenol A dimethacrylate	<10
				ethylene dimethacrylate	<10
				Titanium dioxide	<0.1
				Mequinol; 4-methoxyphenol; hydroquinone monomethyl ether	<0.1

**Table 2:** 3D printing parameters: ASIGA MAX UV PRINTER (385 nm wavelength 3D printer with DLP technology).

No.	Factory Software Parameters	Heater Temperature [°C]	Light Intensity	Burn-In Layers	Burn-In Exposure
1	Gr-17.1 LT (pro3dure)	40	6	1	0.5
2	GR-17 (pro3dure)	30	6	1	0.5
3	GR-10 guide (pro3dure)	30	6	1	0.5

**Table 3:** 3D printing parameters: PHROZEN SHUFFLE LITE PRINTER.

No.	Material	Color	Layer Thickness [mm]	Lowering Speed	Delay [ms]
1	NextDent SG Orange	Orange	0.1	100	1000
2	NextDent C & B MFH	N1	0.05	100	1000
Lower layers					
No.	Material	Number of layers	Exposure time [ms]	Height [mm]	Lifting speed
1	NextDent SG Orange	6	30,000	10	80
2	NextDent C & B MFH	8	40,000	6	80
Normal layers					
No.	Material	Exposure time [ms]	Height [mm]	Lifting speed	
1	NextDent SG Orange	13,500	9	100	
2	NextDent C & B MFH	15,000	4	100	

**Table 4:** Post-fabrication parameters: ASIGA MAX UV PRINTER (385 nm wavelength 3D printer with DLP technology) with post-curing chamber from ASIGA MAX UV PRINTER set.

No.	Factory Software Parameters	Rinsing in Isopropyl Alcohol (min)	Post-Curing (min)
1	Gr-17.1 LT (pro3dure)	4.5	10
2	GR-17 (pro3dure)	4.5	10
3	GR-10 guide (pro3dure)	4.5	10

**Table 5:** Post-fabrication parameters: PHROZEN SHUFFLE LITE PRINTER with post-curing Anycubic Wash & Cure.

No.	Material	Color	Rinsing in Isopropylalcohol (min)	Post-Curing (min)
1	NextDent SG Orange	Orange	4.5	20
2	NextDent C & B MFH	N1	4.5	20

**Table 6:** (**a**) Descriptive statistics of indentation hardness, stiffness and indentation modulus of the surface of the tested materials—materials without aging; (**b**) Descriptive statistics of indentation hardness, stiffness and indentation modulus of the surface of the tested materials—materials after aging.

**(a) Descriptive Statistics of Indentation Hardness, Stiffness and Indentation Modulus of the Surface of the Tested Materials—Materials without Aging**						
**Mechanical Size**	**Statistical Parameter**	**Materials without Aging**				
		**M I**	**M II**	**M III**	**M V**	**M VI**
N (no. of. specim.) = 5	n (no. of. obs.)	25	25	25	25	25
*H_IT_* (O & P) [MPa]	Mean	78.414	84.475	81.767	254.001	116.568
	Std Dev	1.437	1.445	1.795	21.086	5.63
	Min.	76.06	80.087	77.102	211.728	107.606
	Max.	81.377	86.927	85.799	289.394	129.209
	Median	78.207	84.652	82.169	249.701	116.89
	Shape (Weibull)	61.572	67.557	52.147	13.893	23.579
	Scale (Weibull)	79.104	85.153	82.614	263.37	119.18
*S* (O & P) [mN/μm]	Mean	383.728	315.936	372.101	420.71	1170.405
	Std Dev	4.023	2.557	1.971	3.103	126.55
	Min.	374.736	311.198	368.443	414.862	682.258
	Max.	390.423	320.156	377.42	426.686	1394.335
	Median	384.018	315.211	371.749	420.5	1183.031
*E_IT_* (O & P) [GPa]	Mean	1.918	1.639	1.899	3.788	7.16
	Std Dev	0.033	0.023	0.027	0.172	0.757
	Min.	1.87	1.585	1.825	3.433	4.21
	Max.	1.989	1.672	1.953	4.088	8.57
	Median	1.911	1.638	1.9	3.742	7.229
E* (O & P) [GPa]	Mean	2.108	1.801	2.086	4.163	7.868
	Std Dev	0.036	0.025	0.03	0.189	0.832
	Min.	2.055	1.742	2.006	3.772	4.627
	Max.	2.185	1.838	2.146	4.492	9.417
	Median	2.1	1.8	2.088	4.113	7.944
**(b) Descriptive statistics of indentation hardness, stiffness and indentation modulus of the surface of the tested materials—materials after aging**						
**Mechanical size**	**Statistical parameter**	**Materials after aging**				
		**M I AS**	**M II AS**	**M III AS**	**M V AS**	**M VI AS**
N (no. of. specim.) = 5	n (no. of. obs.)	25	25	25	25	25
*H_IT_* (O & P) [MPa]	Mean	51.565	67.722	50.664	219.804	189.726
	Std Dev	2.864	3.767	2.024	5.56	6.904
	Min.	46.261	60.433	46.551	207.193	178.742
	Max.	55.124	72.87	54.427	228.273	203.9
	Median	52.154	68.128	50.89	219.878	188.594
	Shape (Weibull)	20.454	20.66	29.162	46.17	31.090
	Scale (Weibull)	52.889	69.442	51.588	222.37	192.99
*S* (O & P) [mN/μm]	Mean	249.884	256.947	254.125	401.786	481.595
	Std Dev	2.7	5.212	2.931	5.267	5.916
	Min.	245.445	244.751	248.441	393.725	472.935
	Max.	254.98	263.548	260.178	410.869	489.864
	Median	249.783	257.835	253.171	401.018	483.139
*E_IT_* (O & P) [GPa]	Mean	1.011	1.193	1.02	3.366	3.75
	Std Dev	0.038	0.056	0.031	0.07	0.11
	Min.	0.941	1.073	0.956	3.208	3.602
	Max.	1.067	1.268	1.07	3.48	3.956
	Median	1.025	1.199	1.014	3.365	3.753
E* (O & P) [GPa]	Mean	1.112	1.311	1.121	3.698	4.121
	Std Dev	0.042	0.062	0.034	0.077	0.121
	Min.	1.034	1.179	1.05	3.526	3.958
	Max.	1.173	1.394	1.175	3.824	4.348
	Median	1.126	1.318	1.114	3.698	4.124

**Table 7:** Selected parameters of abrasive wear resistance of the tested materials.

Materials	Max. Hertzian Contact Stress [GPa]	Archard’s Wear Coefficient *K* [mm^2^/N]	Specific Wear Rate *k* [mm^3^/Nm]
No aging			
M I	8.46 · 10^−2^	2.59 · 10^−7^	3.3 · 10^−6^
M II	7.61 · 10^−2^	5.77 · 10^−6^	6.83 · 10^−5^
M III	8.403 · 10^−2^	2.42 · 10^−7^	2.96 · 10^−6^
M V	1.291 · 10^−1^	2.52 · 10^−6^	9.94 · 10^−6^
M VI	1.324 · 10^−1^	7.02 · 10^−7^	6.02 · 10^−6^
After aging			
M I AS	6.16 · 10^−2^	1.00 · 10^−7^	1.94 · 10^−6^
M II AS	2.012 · 10^−1^	7.84 · 10^−6^	1.16 · 10^−4^
M III AS	5.521 · 10^−2^	4.03 · 10^−7^	7.96 · 10^−6^
M V AS	1.226 · 10^−1^	2.76 · 10^−6^	1.25 · 10^−5^
M VI AS	1.316 · 10^−1^	1.60 · 10^−5^	8.42 · 10^−5^

## Data Availability

Data sharing is not applicable to this article.

## Appendix A

**Table A1:** Table Wilcoxon Matched Pairs Test (*H_IT_*) Marked tests are significant at *p* < 0.05.

Group vs. Group	T	Z	*p*-Level
M I & M I AS	0.000000	4.372373	0.000012
M II & M II AS	0.000000	4.372373	0.000012
M III & M III AS	0.000000	4.372373	0.000012
M V & M V AS	2.000000	4.318559	0.000016
M VI & M VI AS	0.000000	4.372373	0.000012

**Table A2:** Table Wilcoxon Matched Pairs Test (*E**). Marked tests are significant at *p* < 0.05.

Group vs. Group	T	Z	*p*-Level
M I & M I AS	0.0000	4.372373	0.000012
M II & M II AS	0.0000	4.372373	0.000012
M III & M III AS	0.0000	4.372373	0.000012
M V & M V AS	0.0000	4.372373	0.000012
M VI & M VI AS	0.0000	4.372373	0.000012

**Table A3:** Table Wilcoxon Matched Pairs Test (*Wear*). Marked tests are significant at *p* < 0.05.

Group vs. Group	T	Z	*p*-Level
M I & M I AS	9.00000	1.885695	0.059337
M II & M II AS	0.00000	2.803060	0.005062
M III & M III AS	0.00000	2.803060	0.005062
M V & M V AS	13.00000	1.477977	0.139415
M VI & M VI AS	0.00000	2.803060	0.005062
